# Deferoxamine Treatment Combined With Sevoflurane Postconditioning Attenuates Myocardial Ischemia-Reperfusion Injury by Restoring HIF-1/BNIP3-Mediated Mitochondrial Autophagy in GK Rats

**DOI:** 10.3389/fphar.2020.00006

**Published:** 2020-02-19

**Authors:** Long Yang, Peng Xie, Jianjiang Wu, Jin Yu, Xin Li, Haiping Ma, Tian Yu, Haiying Wang, Jianrong Ye, Jiang Wang, Hong Zheng

**Affiliations:** ^1^ Department of Anesthesiology, The First Affiliated Hospital of Xinjiang Medical University, Urumqi, China; ^2^ Guizhou Key Laboratory of Anesthesia and Organ Protection, Department of Anesthesiology, Zunyi Medical College, Zunyi, China; ^3^ Department of Anesthesiology, Chongqing Health Center for Women and Children, Chongqing, China

**Keywords:** mitochondrial autophagy, sevoflurane postconditioning, diabetes, ischemia-reperfusion, hypoxia-inducible factor-1

## Abstract

Mitochondrial autophagy is involved in myocardial protection of sevoflurane postconditioning (SPostC) and in diabetic state this protective effect is weakened due to impaired HIF-1 signaling pathway. Previous studies have proved that deferoxamine (DFO) could activate impaired HIF-1α in diabetic state to restore the cardioprotective of sevoflurane, while the specific mechanism is unclear. This study aims to investigate whether HIF-1/BNIP3-mediated mitochondrial autophagy is involved in the restoration of sevoflurane postconditioning cardioprotection in diabetic state. Ischemia/reperfusion (I/R) model was established by ligating the anterior descending coronary artery and sevoflurane was administered at the first 15 min of reperfusion. Myocardial infarct size, mitochondrial ultrastructure and autophagosome, ATP content, mitochondrial membrane potential, ROS production, HIF-1α, BNIP3, LC3B-II, Beclin-1, P62, LAMP2 protein expression were detected 2 h after reperfusion, and cardiac function was evaluated by ultrasound at 24 h after reperfusion. Our results showed that with DFO treatment, SPostC up-regulated the expression of HIF-1α and BNIP3, thus reduced the expression of key autophagy proteins LC3B-II, Beclin-1, p62, and increased the expression of LAMP2. Furthermore, it reduced the accumulation of autophagosomes and ROS production, increased the content of ATP, and stabilized the membrane potential. Finally, the myocardial infarction size was reduced and cardiac function was improved. Taken together, DFO treatment combined with SPostC could alleviate myocardial ischemia reperfusion injury in diabetic rats by restoring and promoting HIF-1/BNIP3-mediated mitochondrial autophagy.

## Introduction

Cardiovascular disease (CVD) is a globally prominent social medical problem, and the prevalence rate is rising and becoming the leading cause of death worldwide (van der Ende et al., 2017). Diabetes Mellitus (DM) is one of the most important risk factors of cardiovascular disease (Fox et al., 2015). It is reported that the risk of CVD in diabetic patients increases by 1.38 times every 10 years, and the risk of dying from CVD increased by 1.86 times (Roger et al., 2011). The risk of myocardial ischemia-reperfusion injury in CVD patients with diabetes is 2 to 3 times higher than that of CVD patients without diabetes (Preis et al., 2009), which is the reason of high perioperative mortality in diabetics. Therefore, how to alleviate perioperative myocardial ischemia reperfusion injury of diabetics mellitus is a multidisciplinary problem need to be urgently solved.

Sevoflurane is an inhaled volatile anaesthetic that is widely used in clinical and basic research due to its pharmacological characteristics such as induction of stability and rapid recovery. Many studies have confirmed that sevoflurane postconditioning (SPostC) can alleviate ischemia-reperfusion injury effectively in healthy myocardium (Yu et al., 2015; Wu et al., 2017b; Qiao et al., 2019). However, in the diabetic state, myocardial protection of SPostC is weakened (Drenger et al., 2011; Tai et al., 2012; Gao et al., 2016), and the specific mechanism remains unclear. Our previous study found that HIF-1α is an endogenous target that mediates the protective effects of sevoflurane postconditioning (Yang et al., 2016), but inactivated in diabetic state (Thangarajah et al., 2010; Bento and Pereira, 2011). Therefore, it is proposed that impaired HIF-1α is the reason causing SPostC protection weakening in diabetic state, and in animal experiments it is proved by our research group that cobalt chloride or deferoxamine reversed the impaired HIF-1α and restored the protection of sevoflurane postconditioning (Wu et al., 2017a; Xie et al., 2017).

Mitochondrial autophagy is a defensive metabolic process in which cells adapt to hypoxia. It removes dysfunction mitochondria through autophagy selectively, promotes mitochondrial renewal and recycling, and ensures the stability of mitochondrial function, thereby promoting cell survival (Lemasters, 2005; Saito and Sadoshima, 2015). Previous studies have shown that hypoxia-induced mitochondrial autophagy mostly exists as a protective mechanism, and activated relying on autophagy protein (Gui et al., 2016). BNIP3 is a mitochondrial autophagy receptor related to hypoxia, and is a crucial target downstream gene of HIF-1α, which is considered to be an important signaling molecule for hypoxia-induced mitochondrial autophagy (Ney, 2015). Regulated by HIF-1α, BNIP3 changes the mitochondrial permeability, and promotes mitochondrial autophagy ultimately during myocardial ischemia-reperfusion injury (Ma et al., 2012). However, in the diabetic state, mitochondrial autophagy is inhibited (Kanamori et al., 2015), and the specific reason is unclear. Recently, Zhang reported that mitochondrial autophagy plays an important role in sevoflurane postconditioning to reduce myocardial ischemia-reperfusion injury (Zhang et al., 2014b), but the concrete mechanism has not been elucidated to now. Based on the above research, whether HIF-1α/BNIP3-mediated mitochondrial autophagy is involved in the sevoflurane postconditioning to alleviate myocardial ischemia-reperfusion injury in non-diabetic state remains unclear, and it is also unclear whether the weakened protective effect of SPostC is related to the inhibition of HIF-1α/BNIP3-mediated mitochondrial autophagy in diabetic state.

The purpose of this study was to determine the effect of DFO treatment combined with SPostC on mitochondrial autophagy, and to verify that restoration of HIF-1α/BNIP3-mediated mitochondrial autophagy in diabetic state is the key to restore the protective effect of SPostC.

## Materials and Methods

This study was approved by the First Affiliated Hospital of Xinjiang Medical University Animal Ethics Committees. A total of 280 male Goto-Kakizaki (GK) rats (14–16 weeks old, 300–350g) were purchased from Changzhou Cavans Experimental Animal Co., Ltd. (Certifcate no. SCXK2016-0010). A total of 200 male age-matched Sprague-Dawley (SD) rats were purchased from Changsha Tianqin Biotechnology Co., Ltd. (Certifcate no. SCXK 2014-0011). These animals were kept in rooms maintained at 23 ± 2°C in a 12 h light/dark cycle and were fed a standard rodent diet with free access to water following the Guide for the Care and Use of Laboratory Animals published by the National Institutes of Health (US, 1996 revision).

## Drugs and Reagents

Sevoflurane (Maruishi Pharmaceutical, Japan). Rabbit anti-Beclin polyclonal antibody (ab55878), rabbit anti-LC3B antibody (Sigma, L7543), rabbit anti-BNIP3 monoclonal antibody (ab109414), rabbit anti-HIF-1α monoclonal antibody (abcam, Chip grade), rabbit anti-P62 polyclonal antibody (Ab91526), rabbit anti-LAMP2 monoclonal antibody (AB13524), HIF-1α inhibitor 2ME2 (selleck, USA). Deferoxamine (Novartis Pharma Stein AG, Switzerland). Pentobarbital was purchased from Shanghai Tyrael Biological Technology Co., LTD.

## Myocardial Ischemia-Reperfusion Model

The model of ischemia-reperfusion was simulated by ligation of the anterior descending coronary artery in rats and the experimental procedures were described as previous study (Xie et al., 2017). Briefly, rats were anesthetized with intraperitoneal injection of sodium pentobarbital (50 mg/kg) and fixed to the rat table after the eyelids and pedal reflexes disappeared completely. The laryngoscope was used for tracheal intubation with a 17# trocar, and the rats were mechanically ventilated using a small animal ventilator. The tidal volume was 30–50 ml/kg, the respiratory rate was 60 beats/min, the ratio of inspiration to expiration = 1:2, and the FiO2 was 99%. The anal temperature was maintained at 37°C with a animal insulation blanket, and a biosignal acquisition and processing system was used to continuously monitor the heart rate. An incision was made in the 3rd or 4th intercostal space at the left sternal margin. The individual layer of chest wall muscles was then divided bluntly to access the thoracic cavity, and the pericardium was incised to expose the heart. An ischemia-reperfusion model (40 min ischemia and 2 h reperfusion) was prepared by ligation of the left anterior descending coronary artery (LAD) about 2 mm below the root of the left atrial appendage with a 6/0 silk thread. Myocardial ischemia was confirmed by regional epicardial cyanosis below the level of the ligation and ST-segment elevations in the electrocardiogram.

## Experimental Protocol

### SPostC Promotes HIF-1α/BNIP3 Mediated Mitochondrial Autophagy in Non-Diabetic State

**Figure 1:**
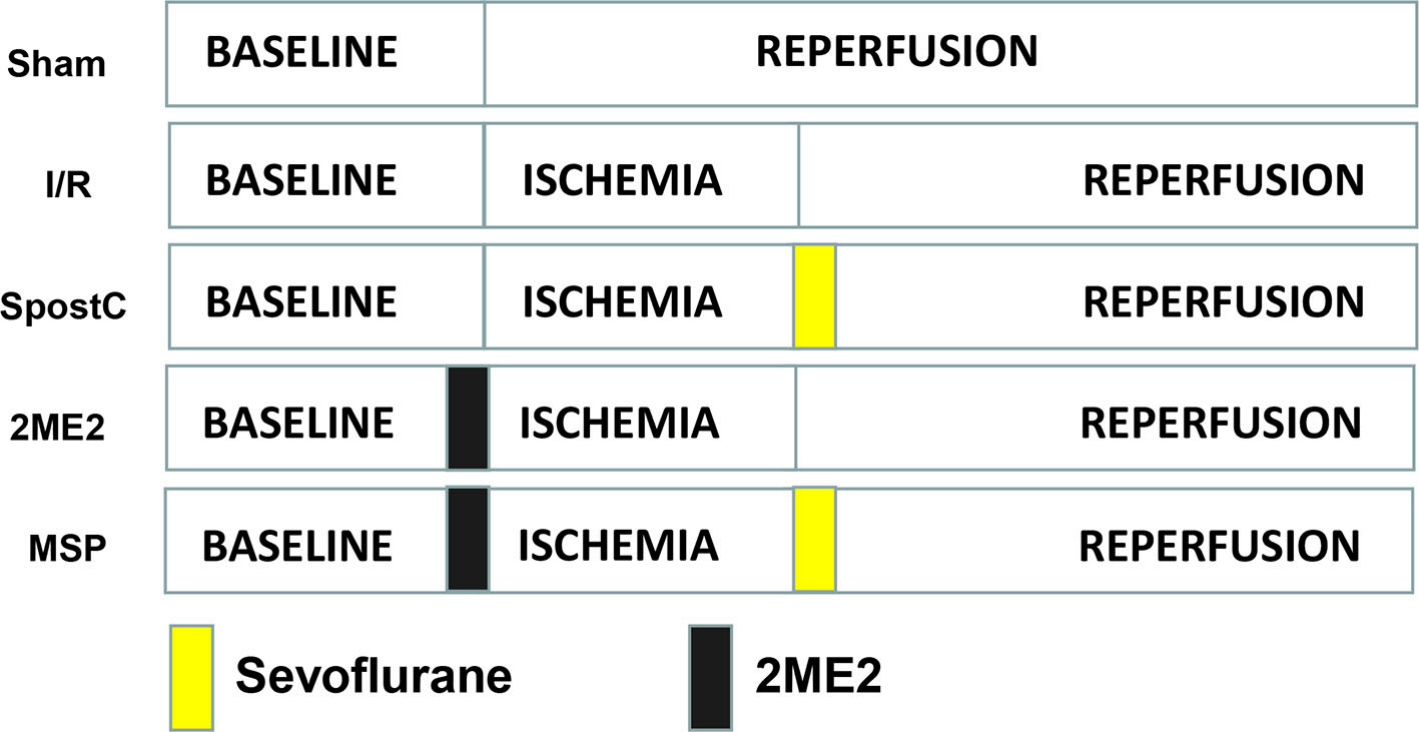
Schematic diagram of ischemia reperfusion in Sprague-Dawley (SD) rats. Except the Sham group, all hearts were subjected to 40 min ischemia, followed by reperfusion for 120 min. The SpostC was executed by administration of 2.4% sevoflurane (1.0 MAC) for 15 min at onset of reperfusion, and then reperfusion for 105 min. MSP and 2ME2 groups were intraperitoneally injected with 2ME2 (15 mg/kg) before ligation of LAD, and the remaining processing is the same as SpostC or I/R groups.

SD rats were randomly divided into the following 5 groups (**Figure 1**): sham group, ischemia/reperfusion (I/R) group, sevoflurane postconditioning (SpostC) group, HIF-1α-selective blocker (2-Methoxyestradiol, 2ME2) + SpostC (MSP) group, 2ME2+ I/R (2ME2) group. Except for the Sham group, the other groups were ligated with LAD for 40 min and then reperfused for 120 min. The SpostC group inhaled 2.4% sevoflurane(1.0 MAC) at the beginning of reperfusion for 15 min and followed by 105 min reperfusion without sevoflurane. MSP and 2ME2 groups were intraperitoneally injected with 2ME2 (15 mg/kg) before ligation of LAD (Mao et al., 2013), and the remaining processing is the same as SpostC or I/R groups.

### DFO Combined With SpostC Against Myocardial Ischemia-Reperfusion Injury in Diabetic State

**Figure 2:**
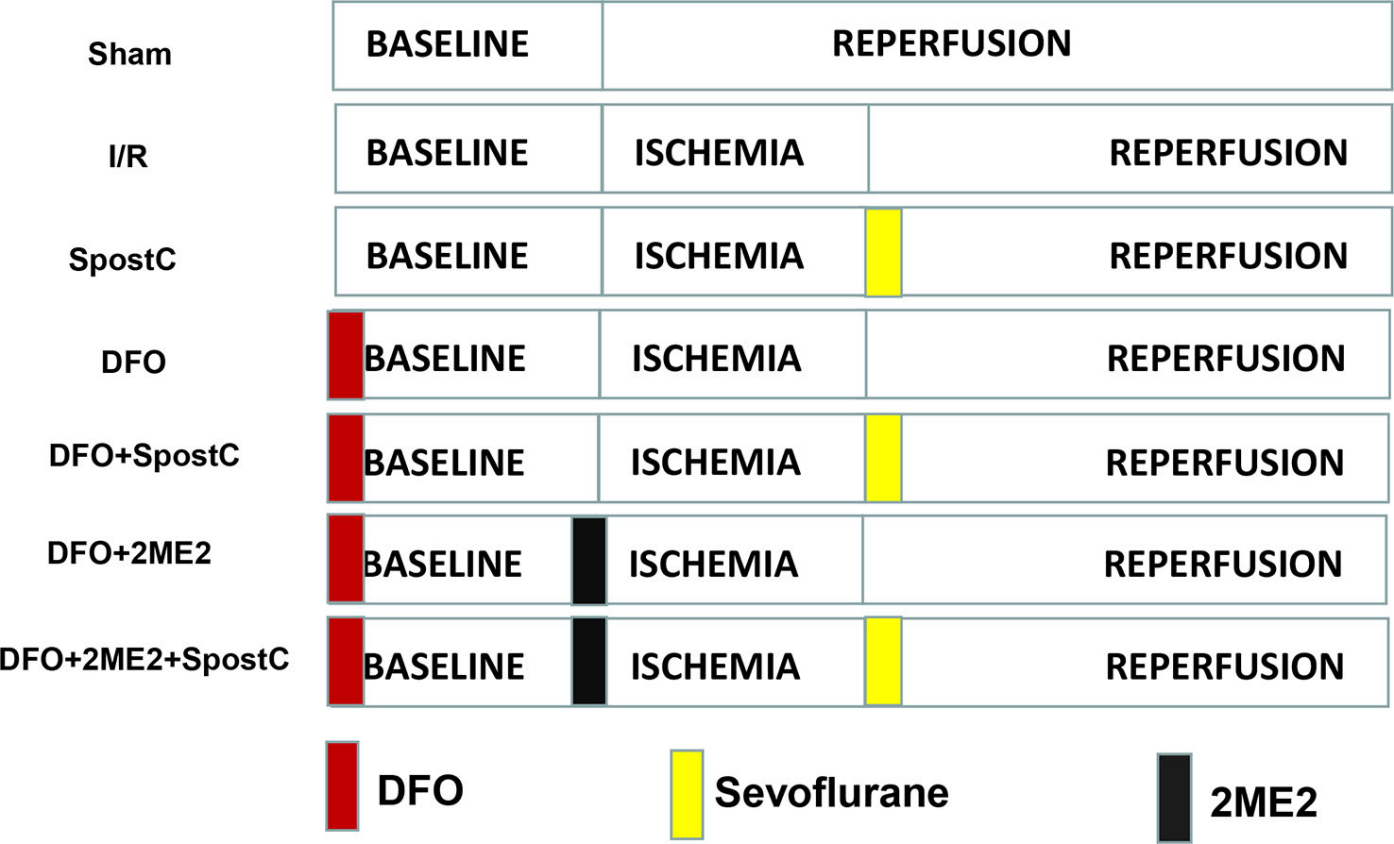
Schematic diagram of ischemia reperfusion in Goto-Kakizaki (GK) rats. Except the Sham group, all hearts were subjected to 40 min ischemia, followed by reperfusion for 120 min. DFO (200 mg/kg) was given intraperitoneally 24 h before the experiment. SpostC and 2ME2 are treated as above (**Figure 1**).

Diabetic GK rats were randomly divided into 7 groups (**Figure 2**): sham group, ischemia/reperfusion (I/R) group, SpostC group, DFO group, DFO+SpostC group, DFO+2ME2+SpostC group, DFO+2ME2 group. Except for the Sham group, the other groups were ligated with LAD for 40 min and then reperfused for 120 min. DFO (200 mg/kg) was given intraperitoneally 24 h before the experiment (Dendorfer et al., 2005). SpostC and 2ME2 were treated as above.

### Mitochondrial Isolation Procedure

At the end of reperfusion, the hearts were collected and homogenized immediately in lysis buffer, and the cytosolic and mitochondrial fractions were separated using a Tissue Mitochondria Isolation kit (Beyotime, C3606, China) following the manufacturer's instructions. The final precipitate was re-suspended in Mitochondrial Extraction Buffer Mix and analyzed as the mitochondrial fraction. The protein concentration of the supernatant was determined using a BCA Protein Assay kit (Beyotime, P0011, China).

### ATP Level Measurement

The ATP level was measured by a firefly luciferase-based ATP assay kit (Beyotime, S0026, China). The assay was operated by the manufacturer's instructions (Zhang et al., 2014a). Briefly, 20 mg myocardial tissue per group were lysed in ATP lysis buffer (200ul), glass homogenization equipment for full homogenization, then they were centrifuged at 12,000 g for 5 min at 4°C, and the supernatant was removed for the ATP assay. ATP detection reagent (100 ul) were added into a microwell for 5 min at room temperature, the samples (20 ul) were then added, and the contents were mixed for 5 sec and measured by a multi-function microplate reader. The ATP concentrations were calculated from standard curve data and expressed as nmol per mg protein. (n = 6/group).

### Analysis of Mitochondrial Membrane Potential (△Ψm)

Mitochondrial membrane potential was examined by a fluorescent probe JC-1 according to the manufacturer's directions (Beyotime, C2006, China) (Meng et al., 2011). Briefly, purified mitochondria 10 ul and 5-fold diluted JC-1 staining solution 90 ul were added to each well of a 96-well cell culture plate, and fluorescence was measured on a multi-function microplate reader at 525 nm excitation/590 nm emission for JC-1 polymer,490nm excitation/530 nm emission for JC-1 monomer, representing the intensity of red and green fluorescence, respectively. Data is expressed as a ratio of red/green Fluorescence Unit (R/G FLU). (n = 6/group).

### ROS Assay Assessment

Fresh left ventricular myocardium was infiltrated with OCT (Sakura, USA) and stored at -80°C for 30 min. 10 μm myocardium sections were cut by a freezing microtome. The prepared sections (10 μm) were washed three times by PBS for 5 min each time. Then, the sections were incubated with ROS Fluorescent Probe-DHE (10 μM, diluted by PBS) at 37°C for 30 min and then washed with PBS as before. Finally, the sections were enclosed with quenching agent and observed with fluorescence microscopy. The whole process needs to avoid light. The numbers of DHE-positive nuclei were measured and statistical analysis in three random vision fields (Wang et al., 2014). (n = 6/group).

### Measurement of Myocardial Infarct Size

Myocardial infarct size was determined by 2,3,5-triphenyl-2H-tetrazolium chloride (TTC) staining. Briefly, hearts were harvested quickly at the end of reperfusion and then frozen at -80°C for 7 min. Then each heart was cut into 5 slices of uniform thickness from the apex to the bottom of the heart and incubated in a 1% TTC solution at 37°C for 25 min. The slices were then fixed in 10% formaldehyde for 24 h. Finally, the heart slices were arranged in order from large to small and photographed with a camera. The infarct size was calculated by ImageJ software. (n = 6/group).

### Transmission Electron Microscopy

To examine the autophagosomes and mitochondrial ultrastructure changes in the myocardium afterI/R, transmission electron microscopy examination was performed as previously described. Briefly, at the end of reperfusion, the heart was immediately harvested. A 1 mm^3^ piece of myocardial tissue was removed from the left ventricle and fixed in a solution of glutaraldehyde in phosphate buffer at 4°C for 24 h. Ultrathin sections (50–80 nm) were cut according to the standard protocol and stained with 1% uranyl acetate and then observed using a transmission electron microscopy. Quantitative morphometric analysis of autophagic vacuoles was performed by a blinded observer. Five rats were assigned in each group. Ten felds were examined for each rat. (n = 5/group).

### Echocardiography Evaluation

Two-dimensional echocardiography measurements were conducted after 2 h of reperfusion. Cardiac function was studied by M-mode echocardiography using the Vevo 2100 Imaging System. The measurement was operated by an independent professional ultrasound technician. M-mode ultrasound was used to acquire all cardiac function parameters in 5 consecutive cardiac cycles. (n = 6/group).

### Western Blot Analysis

The western blotting was performed as described previously (Xie et al., 2017). 2 h after reperfusion, left ventricular tissue was collected and stored in liquid nitrogen. Total protein was extracted from 1 ml of protein lysate and stored in aliquots at -80°C. The protein concentration was quantitatively measured using the BCA method. The protein was separated using sodium dodecyl sulfate-polyacrylamide gel electrophoresis (SDS-PAGE) and then transferred to a PVDF membrane. The membrane temperature was blocked with 5% non-fat milk for 2 h at room temperature. HIF-1α, BNIP3, LC3B, Beclin-1, P62, LAMP2 monoclonal antibodies (diluted 1:1000) were then added and the membrane incubated overnight at 4°C. The secondary antibody (1:10000) was then added and the membrane was incubated for 1 h in the dark. The Odyssey Infrared Imaging System is used to scan PVDF membranes and determine the fluorescence intensity protein bands. The fluorescence intensity ratio of the target protein band to the GAPDH band was used to indicate protein expression levels. (n = 5/group).

### Statistical Analysis

Statistical analysis was performed using the GraphPad Prism 6.0. The data are presented as the mean ± SD. One-way analysis of variance (ANOVA) was used for statistical analyses of data obtained within the same group and between groups, respectively, followed by Tukey's test for multiple comparisons of group means. Significance was accepted when p < 0.05.

## Results

### General Characteristics of Animals

Fasting blood glucose levels (mmol/L), body weight (g), heart weight (g), and heart weight/body weight (g/Kg) were measured to reflect the general characteristics of rats. Compared with gender and age matched SD rats, GK rats showed a significant increase in blood glucose accompanied by weight loss,and the general characteristics of GK rats did not significantly change after DFO administration (**Table 1**).

**Table 1:** Characteristics of Goto-Kakizaki (GK) and Sprague-Dawley (SD) rats.

Characteristics	SD rats	GK rats	GK rats treated with DFO
**Body weight (g)**	350 ± 8	318 ± 10^*^	315 ± 7^*^
**Heart weight (g)**	1.25 ± 0.03	1.16 ± 0.02^*^	1.15 ± 0.04^*^
**Heart to body weight (g/kg)**	2.96 ± 0.07	3.72 ± 0.03^*^	3.67 ± 0.05^*^
**FBG (mmol/L)**	4.8 ± 0.57	15.5 ± 1.06^*^	14.9 ± 1.15^*^

FBG, fasting blood glucose; GK, Goto-Kakizaki *P＜0.05 vs SD rats.

### SPostC Promotes HIF-1/BNIP3-Mediated Mitochondrial Autophagy Against Myocardial Ischemia-Reperfusion Injury in Non-Diabetic State

**Figure 3:**
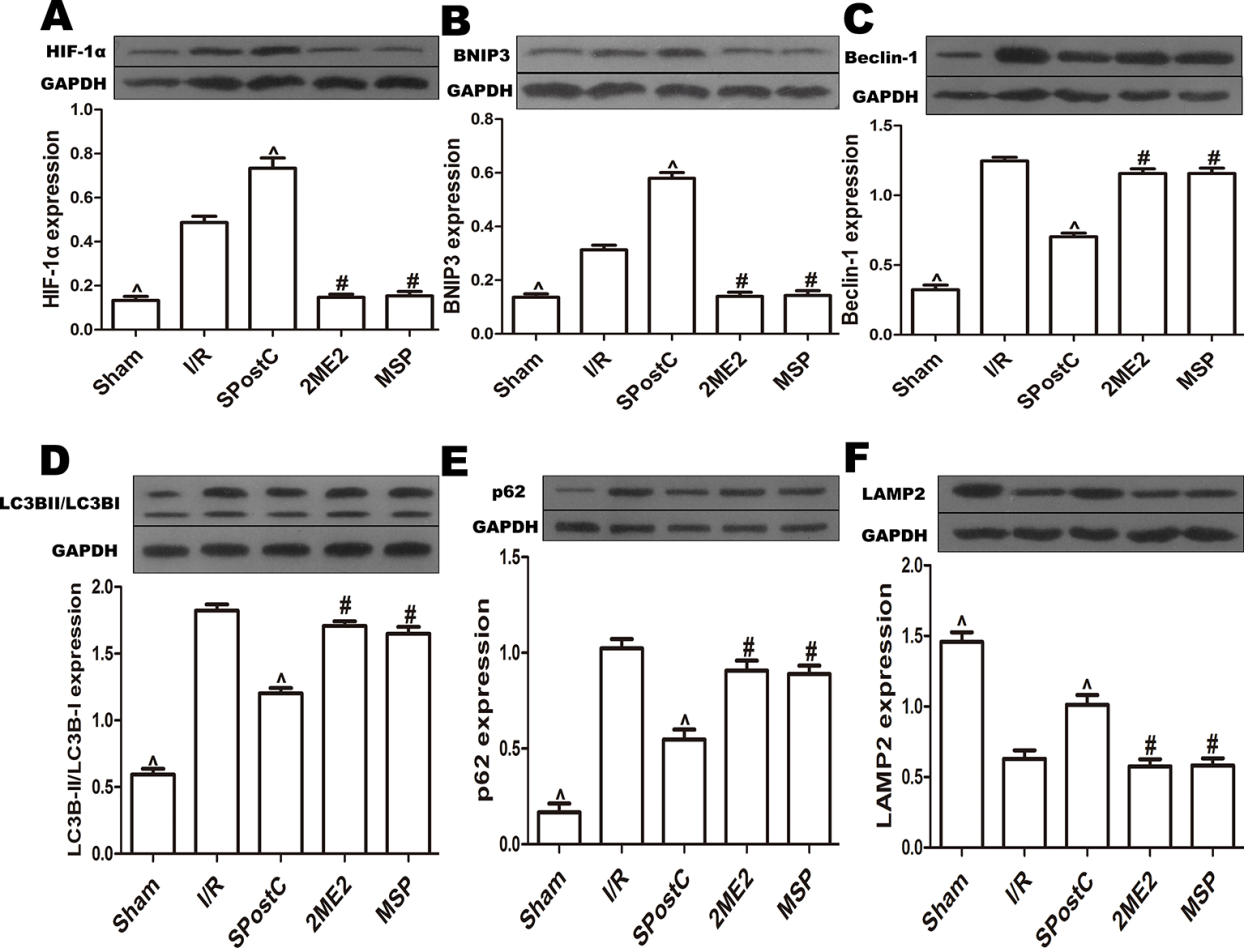
SPostC promoted mitochondrial autophagy by up-regulating HIF-1/BNIP3 signaling pathway protein expression in non-diabetic state. SpostC increased the expression levels of HIF-1α, BNIP3, and LAMP2, while decreased the expression levels of LC3BII, p62 and Beclin-1. **(A)** Expression of HIF-1α. **(B)** Expression of BNIP3. **(C)** Expression of Beclin-1. **(D)** Expression of LC3BII. **(E)** Expression of p62. **(F)** Expression of LAMP2. (^P < 0.05 vs I/R, ^#^P < 0.05 vs SPostC) (n = 5/group).

**Figure 4:**
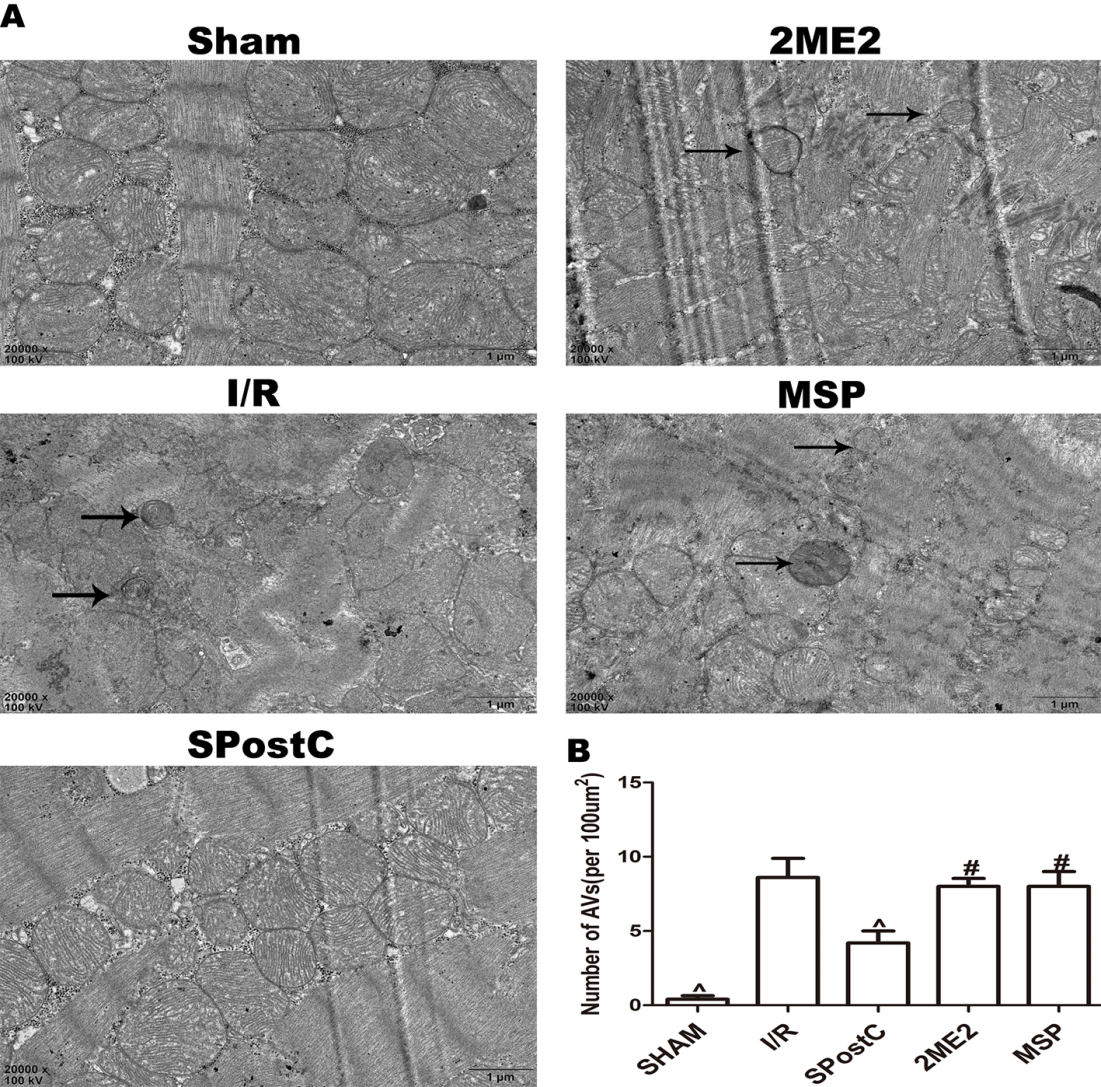
SPostC reduced the damage of mitochondrial ultrastructure and the accumulation of autophagosomes in non-diabetic state. **(A)** Representative transmission electron micrographs show mitochondrial ultrastructure and autophagosomes in the ischemic myocardial after 2 h of reperfusion. **(B)** Quantitative analysis of autophagosome. Autophagosome are indicated by the black arrow. The transmission electron microscope image was magnified 20,000 times. (^P < 0.05 vs I/R, ^#^P < 0.05 vs SPostC) n = 5/group).

Compared with the I/R group, the protein expression levels of HIF-1α, BNIP3 and LAMP2 were significantly increased (P < 0.05 **Figures 3A, B, F**), while LC3BII, P62, and Beclin-1 were significantly decreased (P < 0.05 **Figures 3C–E**) in SPostC group. In addition, as shown in the **Figure 4A** observed by electron microscope, myofilament dissolution, mitochondrial swelling, permutation disorder, interstitial exudation, and accumulation of autophagosomes in I/R group, while in SPostC group, the mitochondrial morphology was basically complete and neatly arranged but a little swelling and the autophagosomes were significantly reduced (P < 0.05 **Figure 4B**). However, the expression of HIF-1α, BNIP3, and LAMP2 protein were significantly decreased (P < 0.05 **Figures 3A, B, F**) after administration of 2ME2 (HIF-1α inhibitor), while the expression of LC3BII, P62 and Beclin-1 were significantly increased (P < 0.05 **Figures 3C–E**). Electron microscopy showed that the level of mitochondrial damage was similar in the 2ME2 group and the MSP group compared with the I/R group, and the autophagosomes were significantly accumulated (P > 0.05 **Figure 4B**).

**Figure 5:**
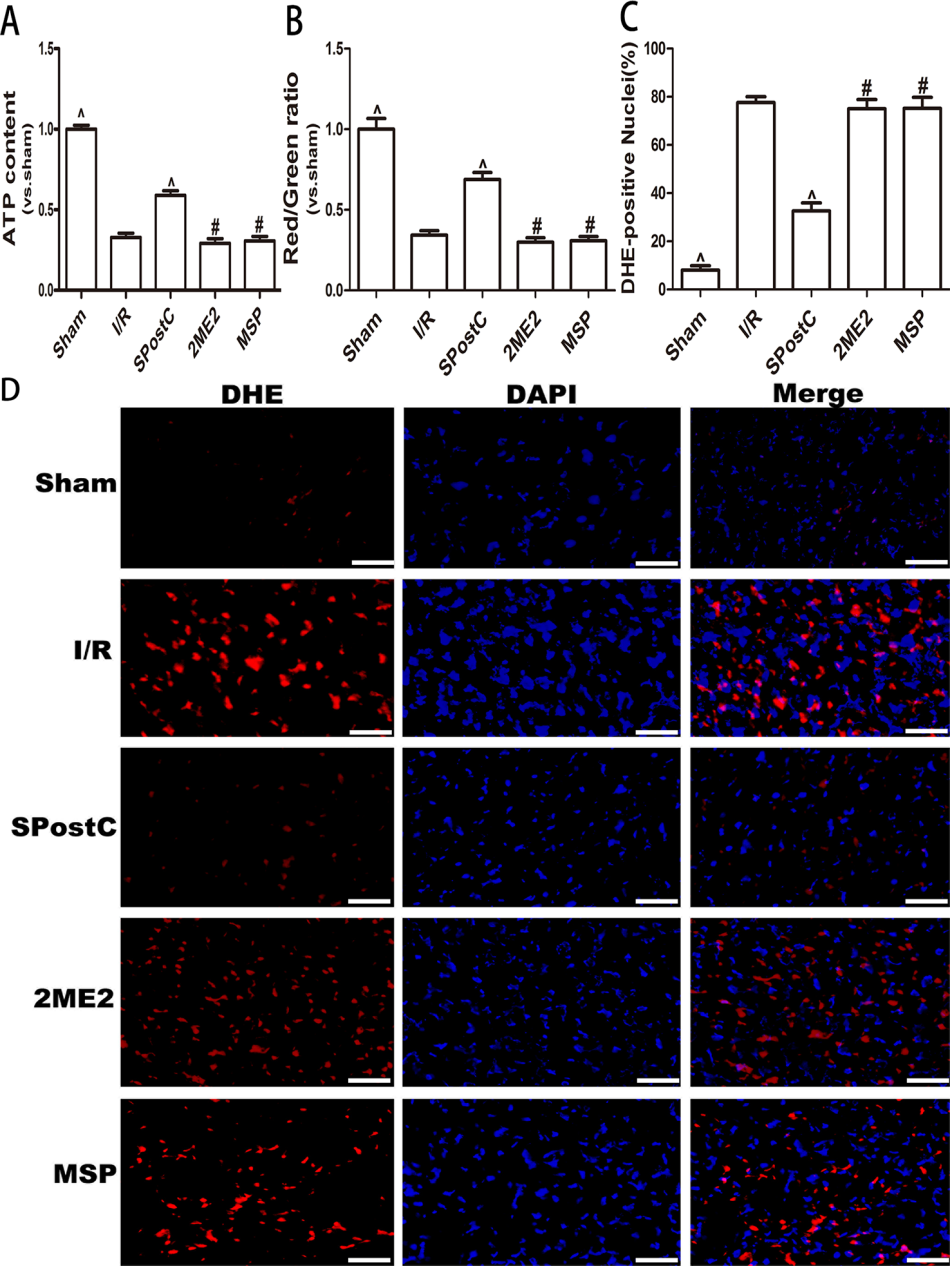
SPostC attenuated myocardial mitochondrial function damage after ischemia-reperfusion in non-diabetic state. SpostC increased ATP content, stabilized membrane potential, and reduced ROS production. **(A)** ATP content. (n = 6/group) **(B)** Mitochondrial membrane potential. (n = 6/group) **(C)** Quantitative analysis of DHE-positive nuclei. **(D)** ROS Fluorescent Probe-DHE. ROS were stained with DHE (red) and nucleus with DAPI (blue). (n = 6/group) (^P < 0.05 vs I/R, ^#^P < 0.05 vs SPostC, Scale bars 50 um).

Compared with the I/R group, ATP content and the membrane potential increased (P < 0.05 **Figures 5A, B**), and the ROS content decreased (P < 0.05 **Figures 5C, D**) in SPostC group; while no significant difference between 2ME2 group and MSP group (P > 0.05 **Figures 5A–D**).

**Figure 6:**
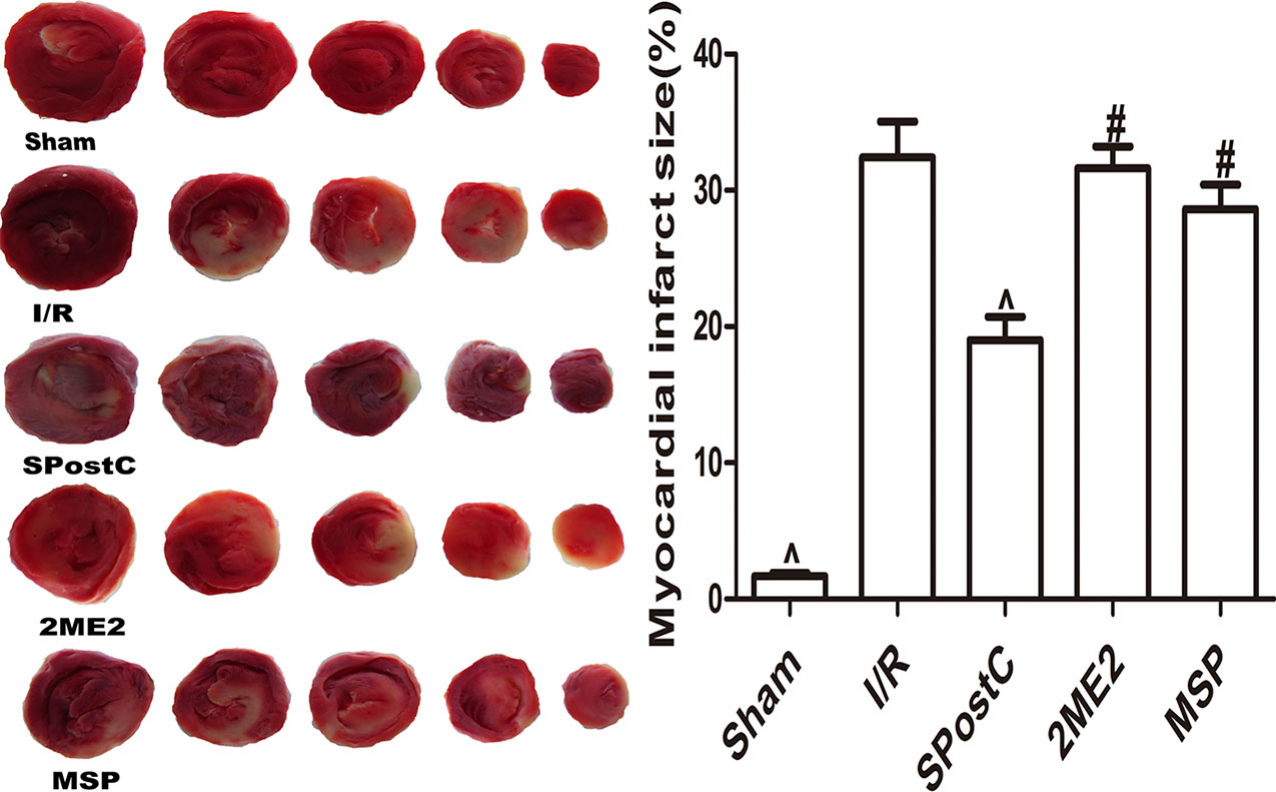
SPostC reduced myocardial infarct size in non-diabetic state. White color represents myocardia infarct area, red color represents the area at risk. The myocardial infarct size was calculated for each slice, and reported as the percent of infarct divided by the total area at risk. (^P < 0.05 vs I/R, ^#^P < 0.05vs SPostC) (n = 6/group).

Compared with the I/R group, the myocardial infarct size was significantly reduced in the SPostC group (P < 0.05 **Figure 6**) while no significant difference between 2ME2 group and MSP group (P > 0.05 **Figure 6**).

**Figure 7:**
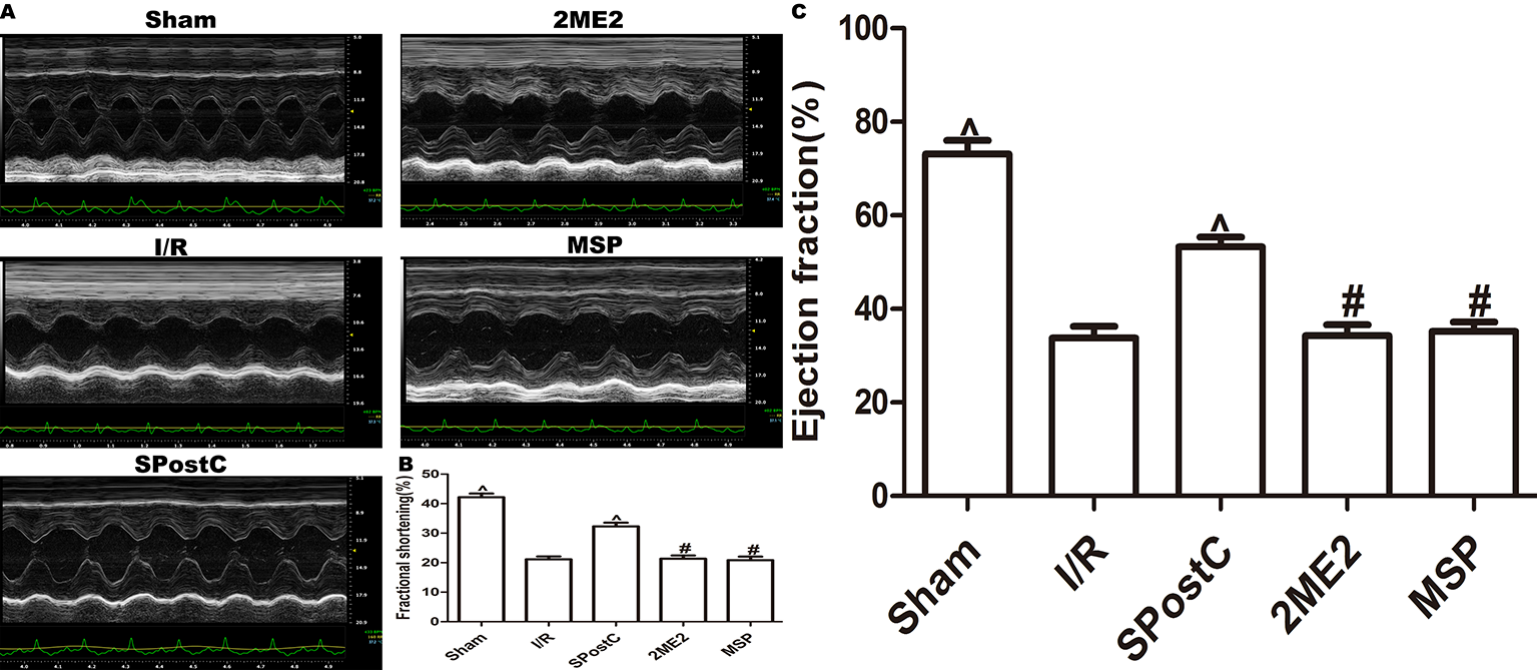
SPostC improved cardiac function after ischemia-reperfusion in non-diabetic state. **(A)** Representative M-mode images of echocardiography. **(B)** FS, fractional shortening. **(C)** EF, ejection fraction. (^P < 0.05 vs I/R, ^#^P < 0.05 vs SPostC) (n = 6/group).

Compared with the sham group, EF, FS, LVAWd, LVAWs, LVPWd, and LVPWs were significantly decreased, while LVIDd and LVIDs were increased (P < 0.05 **Figure 7**, **Table 2**) in the I/R group. Compared with the I/R group, SPostC significantly improved the above indicators. However, there was no significant difference between the 2ME2 group and the MSP group after administration of 2ME2 (P > 0.05 **Figure 7**, **Table 2**).

**Table 2:** Cardiac function indicators in non-diabetic status.

Cardiac function	Sham	I/R	SPostC	2ME2	MSP
**EF(%)**	73.17 ± 7.08^*^	33.83 ± 6.05	53.33 ± 5.05^*^	34.33 ± 5.61	35.17 ± 5.00^#^
**FS(%)**	42.17 ± 3.13^*^	21.17 ± 2.32	32.33 ± 3.01^*^	21.33 ± 2.66	20.83 ± 2.99^#^
**LVIDd(mm)**	4.43 ± 0.29^*^	6.56 ± 0.40	4.93 ± 0.16^*^	6.25 ± 0.16	6.52 ± 0.15^#^
**LVIDs(mm)**	2.6 ± 0.06^*^	4.84 ± 0.11	3.41 ± 0.37^*^	4.37 ± 0.49	4.57 ± 0.40^#^
**LVAWd(mm)**	3.38 ± 0.24^*^	2.16 ± 0.14	3.00 ± 0.28^*^	2.16 ± 0.19	2.14 ± 0.12^#^
**LVAWs(mm)**	4.43 ± 0.10^*^	3.33 ± 0.11	3.95 ± 0.18^*^	3.34 ± 0.13	3.33 ± 0.32^#^
**LVPWd(mm)**	2.83 ± 0.14^*^	1.97 ± 0.25	2.62 ± 0.17^*^	1.95 ± 0.21	1.97 ± 0.22
**LVPWs(mm)**	4.38 ± 0.20^*^	2.42 ± 0.19	3.36 ± 0.34^*^	2.46 ± 0.28	2.38 ± 0.21^#^

*P < 0.05 vs I/R group; ^#^P < 0.05 vs SPostC group. EF, ejection fraction; FS, fractional shortening; LVIDd, left ventricular internal diameter in diastole; LVIDs, left ventricular internal diameter in systole; LVAWd, Left ventricular anterior wall in diastole; LVAWs, Left ventricular anteriorwall in systole; LVPWd, left ventricular posterior wall thickness in diastole; LVPWs, left ventricular posterior wall thickness in systole.

### DFO Combined With SPostC Restored HIF-1/BNIP3-Mediated Mitochondrial Autophagy by Reversing Impaired HIF-1α, and Restored the Protective Effect of SPostC in Diabetic State

**Figure 8:**
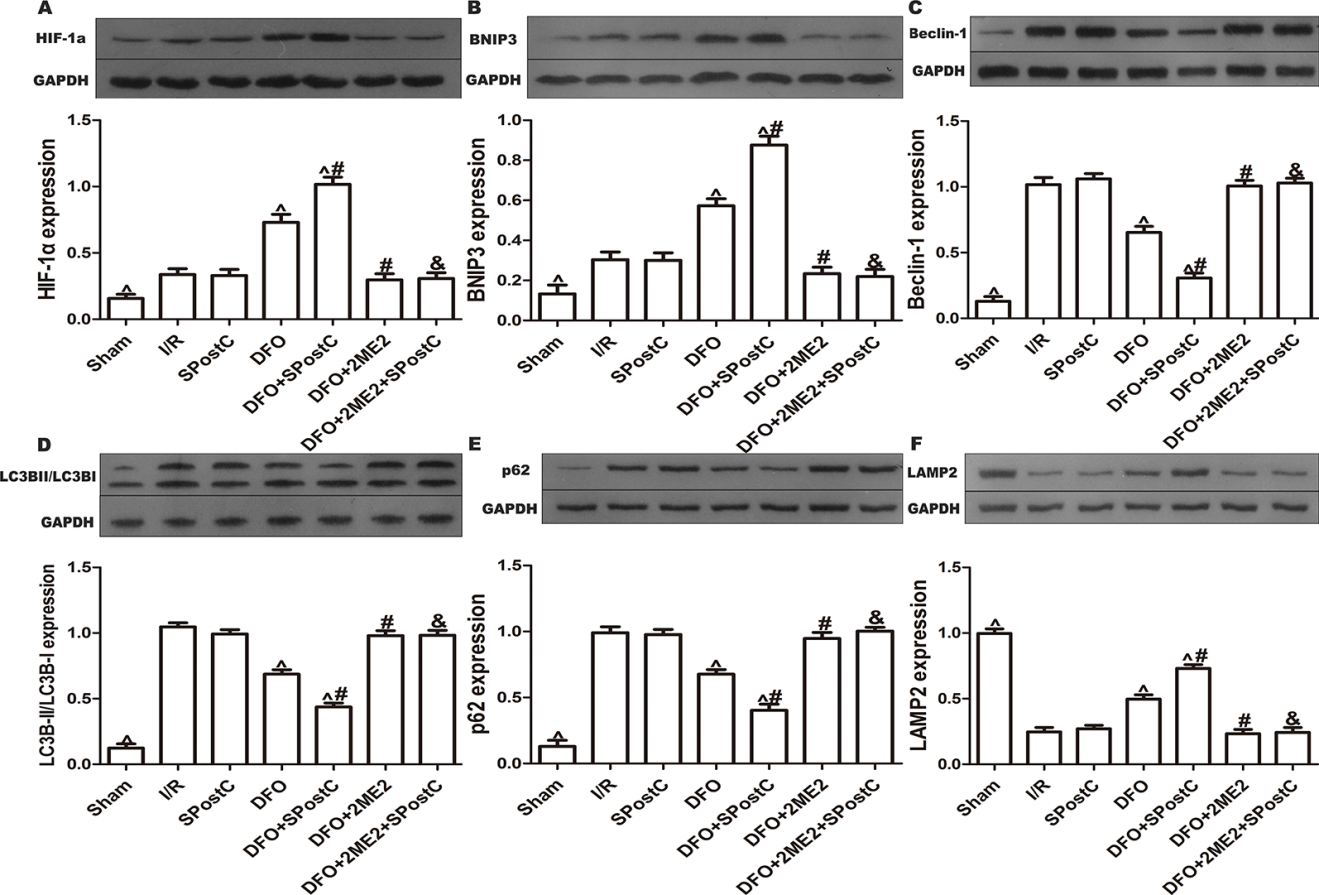
After deferoxamine (DFO) treatment, SPostC promoted mitochondrial autophagy by up-regulating HIF-1/BNIP3 signaling pathway protein expression in diabetic state. SpostC increased the expression levels of HIF-1α, BNIP3 and LAMP2, while decreased the expression levels of LC3BII, p62, and Beclin-1. **(A)** Expression of HIF-1α. **(B)** Expression of BNIP3. **(C)** Expression of Beclin-1. **(D)** Expression of LC3BII. **(E)** Expression of p62. **(F)** Expression of LAMP2. (^P < 0.05 vs I/R, ^#^P < 0.05 vs DFO, ^&^P < 0.05 vs DFO+SpostC) (n = 5/group).

**Figure 9:**
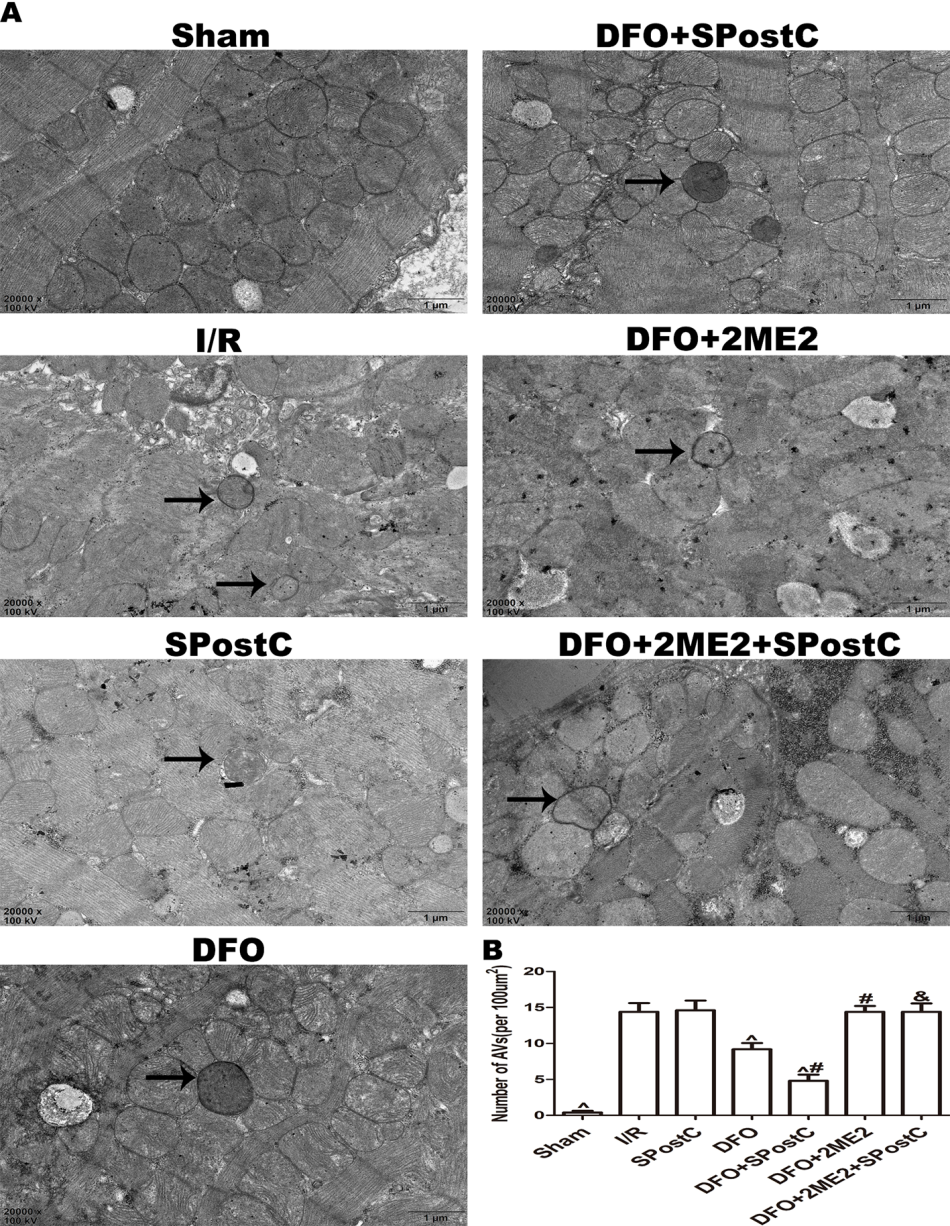
After deferoxamine (DFO) treatment, SPostC reduced the damage of mitochondrial ultrastructure and the accumulation of autophagosomes in diabetic state. **(A)** Representative transmission electron micrographs show mitochondrial ultrastructure and autophagosomes in the ischemic myocardial after 2 h of reperfusion. **(B)** Quantitative analysis of autophagosome. Autophagosome are indicated by the black arrow. The transmission electron microscope image was magnified 20,000 times. (^P < 0.05 vs I/R, ^#^P < 0.05 vs DFO, ^&^P < 0.05 vs DFO+SpostC) (n = 5/group).

We further examined the protein expression of HIF-1α, BNIP3, LC3BII, P62, Beclin-1, LAMP2, and there is no significant difference between SPostC group and I/R group (P > 0.05 **Figures 8A–F**). As shown in the **Figure 9A**, obvious mitochondria swelling, widened or broken cristae gaps, highly dilated sarcoplasmic reticulum, dissolved or ruptured filaments, and accumulation of autophagosomes were observed in the I/R group, SPostC group, DFO+2ME2+SPostC group and DFO+2ME2 (P > 0.05 **Figure 9B**). After administration of DFO, the expression of HIF-1α, BNIP3, and LAMP2 protein levels were increased (P < 0.05 **Figures 8A, B, F**), while LC3BII, P62, and Beclin-1 protein levels were decreased (P < 0.05 **Figures 8C–E**) in the DFO+SPostC group. In addition, the mitochondria morphology was almost complete and neatly arranged, except a slight swelling, and the autophagosomes were significantly reduced in the DFO+SPostC group (P < 0.05 **Figure 9B**).

**Figure 10:**
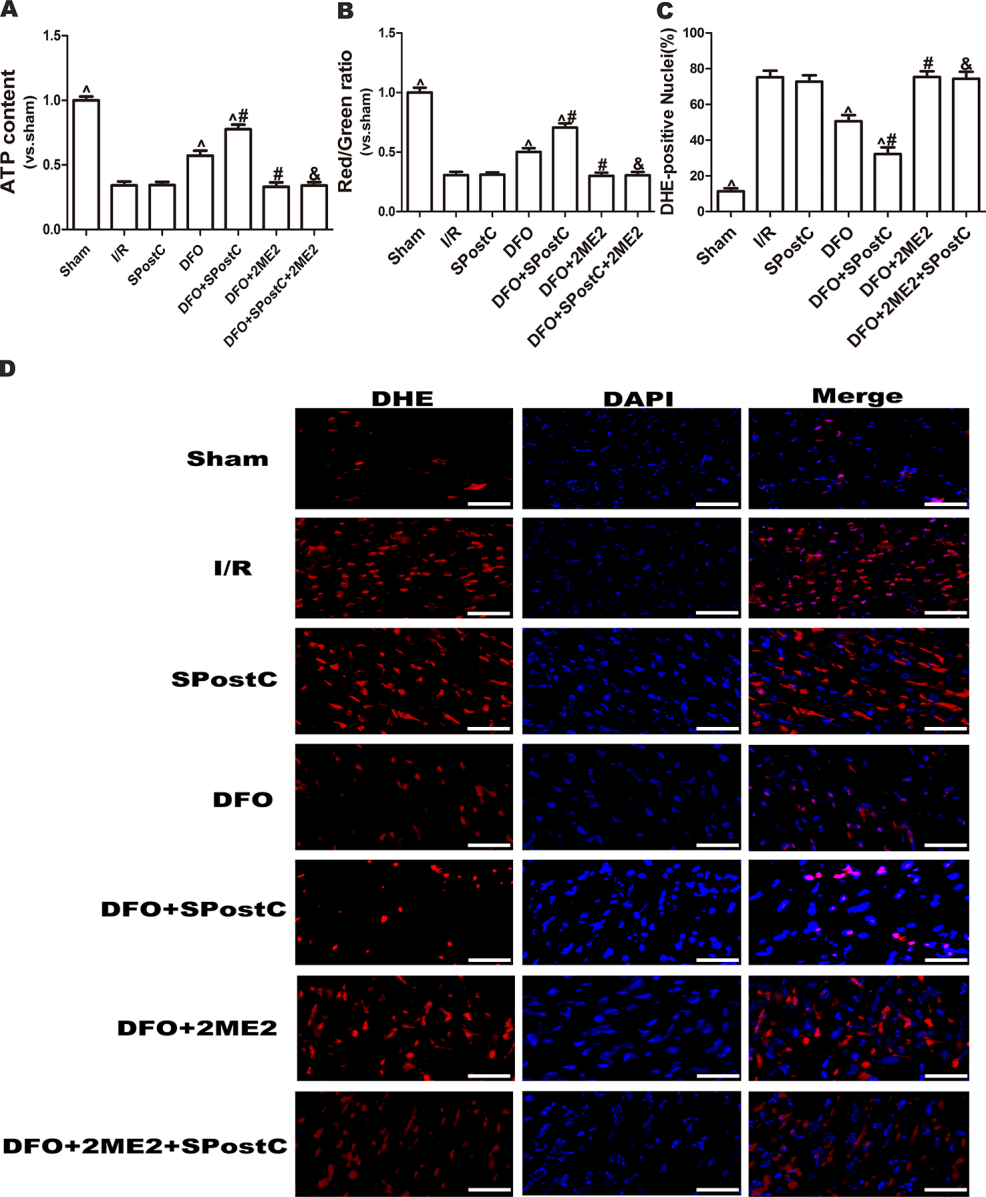
After deferoxamine DFO treatment, SPostC attenuated myocardial mitochondrial function damage after ischemia-reperfusion in diabetic state. SpostC increased ATP content, stabilized membrane potential, and reduced ROS production after DFO treatment. **(A)** ATP content. (n = 6/group) **(B)** Mitochondrial membrane potential. (n = 6/group) **(C)** Quantitative analysis of DHE-positive nuclei. **(D)** ROS Fluorescent Probe-DHE. ROS were stained with DHE (red) and nucleus with DAPI (blue). (n = 6/group) (^P < 0.05 vs I/R, ^#^P < 0.05 vs DFO, ^&^P < 0.05 vs DFO+SpostC, Scale bars 50um).

As shown in the **Figure 10**, ATP content and membrane potential significantly decreased, while ROS production increased in I/R group, SPostC group, DFO+2ME2+SPostC group, DFO+2ME2 group (P > 0.05 **Figure 10**). After DFO administration, ATP content and membrane potential significantly increased (P < 0.05 **Figures 10A, B**), while ROS production decreased (P < 0.05 **Figures 10C, D**) in DFO+SPostC group.

**Figure 11:**
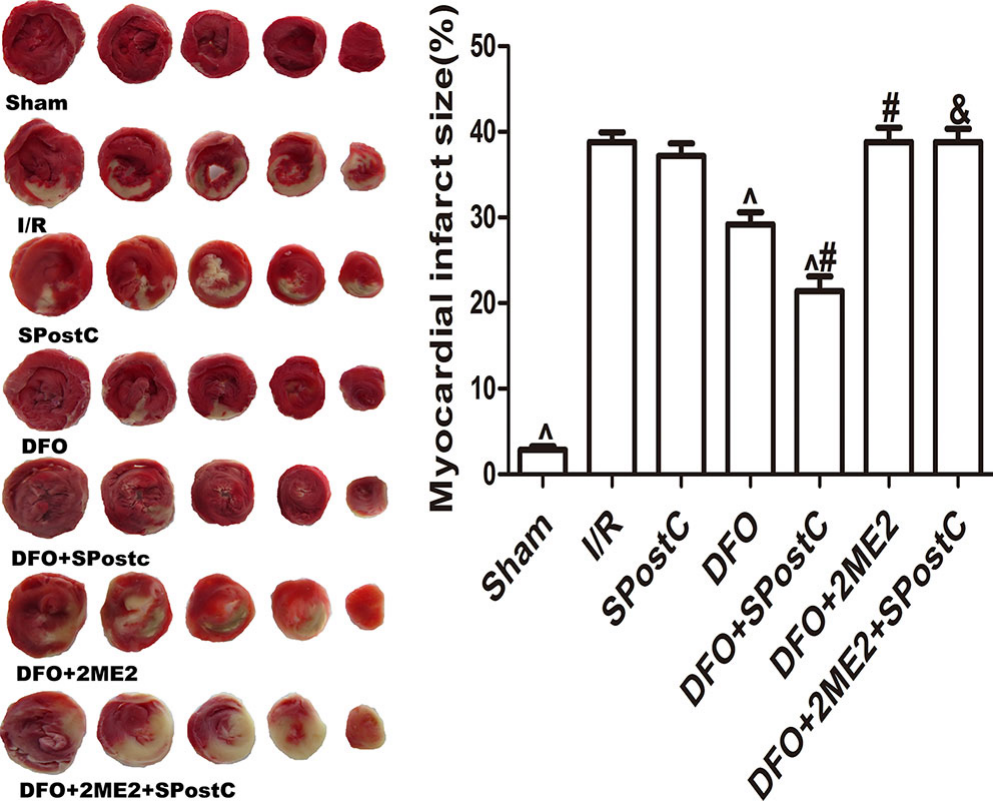
After deferoxamine (DFO) treatment, SPostC reduced myocardial infarct size in diabetic state. White color represents myocardia infarct area, red color represents the area at risk. The myocardial infarct size was calculated for each slice, and reported as the percent of infarct divided by the total area at risk. (^P < 0.05 vs I/R, ^#^P < 0.05 vs DFO, ^&^P < 0.05 vs DFO+SpostC) (n = 6/group).

As shown in the **Figure 11**, compared with the I/R group, SPostC did not reduce myocardial infarct size (P > 0.05 vs. I/R). After DFO treatment, myocardial infarct size decreased significantly in DFO+SPostC group (P < 0.05 vs. I/R, SPostC,DFO). However, after 2ME2 administration, the myocardial protection of DFO combined with SPostC disappeared, and myocardial infarct size was no significant difference among DFO+2ME2+SPostC group, DFO+2ME2 group and I/R group (P > 0.05 **Figure 11**).

**Figure 12:**
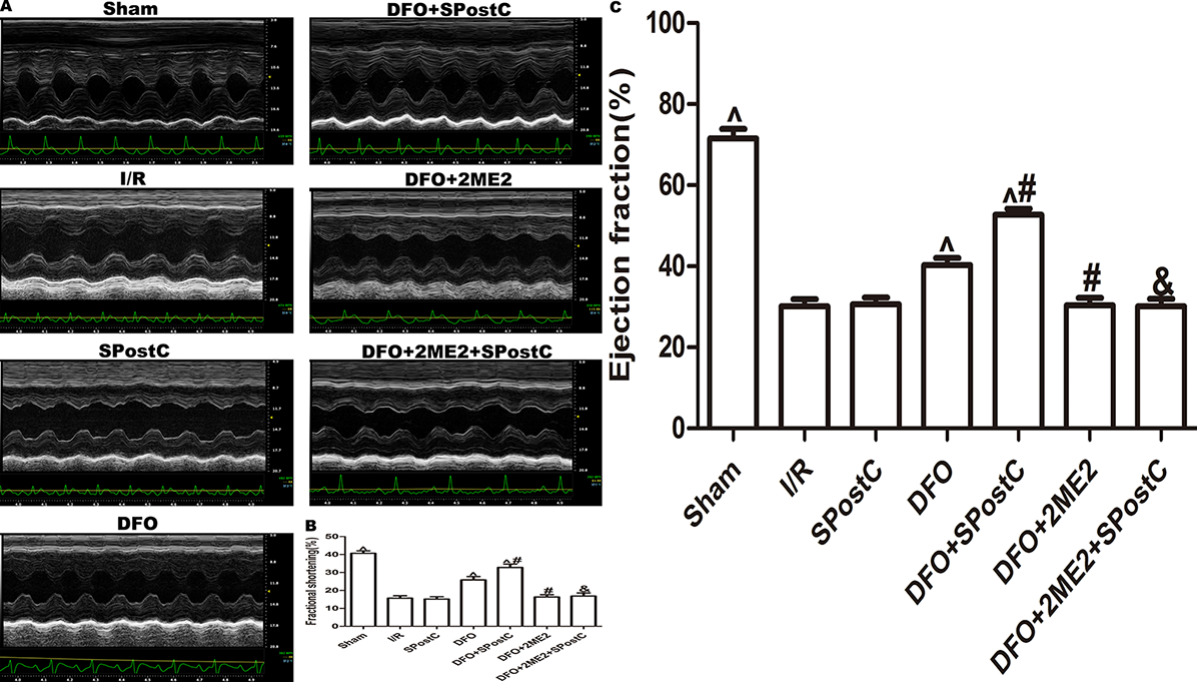
After deferoxamine (DFO) treatment, SPostC improved cardiac function after ischemia-reperfusion in diabetic state. **(A)** Representative M-mode images of echocardiography. **(B)** FS, fractional shortening. **(C)** EF, ejection fraction. (^P < 0.05 vs I/R, ^#^P < 0.05 vs DFO, ^&^P < 0.05 vs DFO+SpostC) (n = 6/group).

As shown in the **Figure 12** and **Table 3**, compared with the sham group, EF, FS, LVAWd, LVAWs, LVPWd, and LVPWs were significantly decreased, while LVIDd and LVIDs were increased in I/R group(P < 0.05). There was no significant difference in the above indicators between SPostC group and I/R group (P > 0.05). However, the above indicators were significantly improved after DFO treatment (P < 0.05). In contrast, after 2ME2 administration, the cardioprotective effects of DFO+SPostC were abolished (P > 0.05).

**Table 3:** Cardiac function indicators in diabetic status.

Cardiac function	Sham	I/R	SPostC	DFO	DFO+SPostC	DFO+2ME2	DFO+2ME2+SPostC
**EF(%)**	71.5 ± 5.89^*^	30.17 ± 4.17	30.67 ± 3.98	40.33 ± 4.13^*^	52.67 ± 3.72^*^^#^	30.33 ± 4.63^#^	30.17 ± 4.31^&^
**FS(%)**	40.67 ± 3.27^*^	15.67 ± 3.27	15.17 ± 3.19	25.83 ± 4.71^*^	32.83 ± 4.54*^#^	16.33 ± 3.08^#^	16.83 ± 4.36^&^
**LVIDd(mm)**	6.05 ± 0.34^*^	8.41 ± 0.16	8.28 ± 0.27	7.18 ± 0.08^*^	6.32 ± 0.17*^#^	8.27 ± 0.23^#^	8.57 ± 0.11^&^
**LVIDs(mm)**	4.25 ± 0.33^*^	7.04 ± 0.26	7.02 ± 0.30	6.02 ± 0.37^*^	5.29 ± 0.23*^#^	7.04 ± 0.18^#^	7.03 ± 0.33^&^
**LVAWd(mm)**	2.59 ± 0.05^*^	1.32 ± 0.02	1.34 ± 0.04	1.57 ± 0.09^*^	2.07 ± 0.05*^#^	1.31 ± 0.03^#^	1.29 ± 0.06^&^
**LVAWs(mm)**	3.69 ± 0.08^*^	2.37 ± 0.04	2.35 ± 0.04	2.93 ± 0.05^*^	3.44 ± 0.06*^#^	2.36 ± 0.08^#^	2.35 ± 0.03^&^
**LVPWd(mm)**	1.75 ± 0.06^*^	1.07 ± 0.04	1.04 ± 0.03	1.20 ± 0.05^*^	1.60 ± 0.07*^#^	1.09 ± 0.02^#^	1.06 ± 0.07^&^
**LVPWs(mm)**	2.65 ± 0.02^*^	1.36 ± 0.04	1.30 ± 0.04	1.85 ± 0.06^*^	2.14 ± 0.04*^#^	1.31 ± 0.03^#^	1.34 ± 0.04^&^

*P < 0.05 vs I/R group; ^#^P < 0.05 vs DFO group; ^&^P < 0.05 vs DFO+SpostC group. EF, ejection fraction; FS, fractional shortening; LVIDd, left ventricular internal diameter in diastole; LVIDs, left ventricular internal diameter in systole; LVAWd, Left ventricular anterior wall in diastole; LVAWs, Left ventricular anteriorwall in systole; LVPWd, left ventricular posterior wall thickness in diastole; LVPWs, left ventricular posterior wall thickness in systole.

## Discussion

The results of this study indicate that SPostC alleviates myocardial ischemia-reperfusion injury *via* promoting HIF-1/BNIP3-mediated mitochondrial autophagy in non-diabetic state. In addition, this study demonstrates that, DFO treatment combined with SPostC in diabetic state can activate impaired HIF-1α and up-regulate HIF-1α protein expression, further promote HIF-1/BNIP3-mediated mitochondrial autophagy and promptly remove dysfunction mitochondria. As a result, damaged mitochondrial-derived ROS was reduced, ATP content was increased, mitochondrial membrane potential was stabilized. Finally, myocardial infarct size was reduced, and cardiac function was improved.

Our previous studies have confirmed that cobalt chloride or deferoxamine can reverse the impaired HIF-1α in diabetic state and restore the protective effect of SPostC (Wu et al., 2017a; Xie et al., 2017). Although the specific molecular mechanism is still unclear, we found that myocardial energy metabolism disorder is the key to the decline of diabetic myocardium against I/R injury and increased vulnerability, while functional integrity of mitochondria are the source of sufficient myocardial motility. Mitochondria are not only the effectors of energy metabolism centers and various survival signals in cells, but also the “targets” of reactive oxygen species attack, which are very sensitive to cell death signals caused by I/R injury (Machado et al., 2009). I/R injury leads to increased ROS level, decreased ATP level, decreased mitochondrial transmembrane potential, while accompany with disorder levels of metabolites and Ca^2+^. Low levels of ATP or transmembrane potential in cells slow down the process by which proteins are transported into the mitochondria, leading to protein imbalance and mitochondrial precursor protein accumulation in the cytoplasm. Damaged mitochondria produce more ROS 10 times than normal mitochondria, and large amounts of ROS can directly attack mitochondrial proteins and DNA, aggravating mitochondrial dysfunction, and thus forming a vicious circle (Elrod and Molkentin, 2013). Therefore, breaking this vicious circle and clearing damaged mitochondria in time is the key to against I/R injury in diabetic myocardium.

Mitochondrial autophagy is first proposed in 2005, which means selectively removes damaged or dysfunctional mitochondria through autophagy pathway to maintain the balance of mitochondrial quality and quantity in the cell, thereby maintaining cell homeostasis (Lemasters, 2005). Recently, the regulation mechanism of mitochondrial autophagy is mainly focused on PTEN-induced putative kinase 1 (PINK1)-Parkin pathway (Wang et al., 2017) and receptor (BNIP3, NIX, FUNDC1) mediated pathways (Jin et al., 2018). BNIP3 is not only a key receptor for mitochondrial autophagy, but also a downstream target gene of HIF-1α (Liu et al., 2019). When cells are subjected to hypoxia, HIF-1 activates downstream *BNIP3* gene transcription and then promotes mitochondrial autophagy to eliminate dysfuction mitochondria (Bellot et al., 2009). The main mechanism is that BNIP3 binds to Bcl-2/Bcl-XL competitively with Beclin-1 *via* BH3 structure, resulting in the release of Beclin-1 out from Bcl-2/Bcl-XL complex, free Beclin-1 can form a PI3K complex together with a variety of proteins, which activates the PI3K/Akt pathway to regulate downstream autophagy-associated ATG proteins, thereby activating mitochondrial autophagy (Kim et al., 2013; Jimenez et al., 2014). In addition, BNIP3 can also directly bind to LC3 to promote mitochondrial autophagy (Hanna et al., 2012).

Ischemic postconditioning is an effective method to reduce organ ischemia-reperfusion injury (Chu et al., 2019; Shao et al., 2019; Zhang et al., 2019). Studies showed that ischemic postconditioning protected the heart from I/R injury by promoting autophagy (Hao et al., 2017). However, the protective effect of ischemic postconditioning is weakened in diabetic state (Bayrami et al., 2018). From clinical practice, SPostC is easier to control than ischemic postconditioning and can exert similar myocardial protective effects as ischemic postconditioning (Chen et al., 2008; Li et al., 2008). Studies have shown that in non-diabetic conditions, SPostC promotes autophagic flux against myocardial ischemia-reperfusion injury through a NO-dependent mechanism, but the effect of SPostC on mitochondrial autophagy and related receptors was not mentioned (Qiao et al., 2019). Zhang et al. found that SPostC promoted autophagic flow against myocardial I/RI by reducing the accumulation of autophagosomes, but the role of signaling pathways that mediate autophagy was not discussed further (Zhang et al., 2014b). Our study found that SPostC can promote mitochondrial autophagy, reduce autophagosome accumulation, stabilize mitochondrial function, and improve cardiac function after I/R. This is consistent with the results of the Zhang's study. However, these protective effects disappeared after 2ME2 administration. The results indicate that HIF-1/BNIP3-mediated mitochondrial autophagy is involved in SPostC against myocardial ischemia-reperfusion injury.

Studies have confirmed that autophagy is a double-edged sword. Excessive autophagy will self-digest or degrade certain important organelles or proteins. Insufficient autophagy can neither remove damaged and aging organelles in time, nor provide raw materials for cell renewal (Sciarretta et al., 2015). Although studies have shown that autophagy is inhibited in type 2 diabetes (Kanamori et al., 2015), specific reasons have rarely been reported. In this study, it was found that in the diabetic state, myocardial I/R injury led to increased expression of LC3II, beclin-1, and p62 protein, decreased expression of LAMP2 protein. In addition, mitochondrial structure swelling and autophagosome accumulation were observed under electron microscope. Although autophagy flux was not observed dynamically, our experimental results were consistent with Bayrami et al., indicating that autophagy flux was impaired in diabetic myocardium after I/R injury. Therefore, how to remove dysfuction mitochondria in time by restoring autophagic flux is particularly important for alleviating myocardial I/RI in diabetic state. Currently, relevant studies have found that vildagliptin combined with IPostC can restore mitochondrial membrane potential and mitochondrial function, and promote autophagy against diabetic myocardium I/RI, but it has not been clarified whether the signaling pathway mediating autophagy plays a role in it (Bayrami et al., 2018). In addition, there is no clear evidence of the effect of SPostC on mitochondrial autophagy after I/RI in diabetic myocardium.

DFO, a highly selective iron chelator, can prevent the hydroxylation of HIFα isoform by inhibiting prolyl hydroxylase, thereby simulating the effect of hypoxia on the cellular oxygen sensor. Studies have shown that systemic or local application of DFO can stabilize and activate HIF-1α, and then reverse the damaged HIF-1 signaling pathway, thereby improving wound healing in diabetic subjects (Hou et al., 2013; Duscher et al., 2015). In this study, we used DFO to activate and stabilize impaired HIF-1 in the diabetic state and found that DFO combined with SPostC significantly promoted the clearance of mitochondrial autophagosomes, reduced ROS production, increased ATP production, stabilized membrane potential, and ultimately reduced myocardial infarct size and improved cardiac function. The protective effect of DFO combined with SPostC is better than using DFO alone, but abolished by 2ME2. The results indicate that HIF-1/BNIP3-mediated mitochondrial autophagy is the key to restore SPostC protection in diabetic state. Moreover, DFO combined with SPostC can restore mitochondrial autophagy mediated by HIF-1/BNIP3 signaling pathway, clear dysfuction mitochondria in time, avoid mitochondria-derived ROS attack on normal mitochondria, and ensure the stability of mitochondrial function.

There are still some limitations in this study. We simply used drugs to intervene the HIF-1 signaling pathway without using gene silencing or viral transfection techniques. Further experiments are also required to verify how to transform the hypothesis into clinical application.

## Conclusions

The study demonstrated that DFO treatment combined with SPostC can activate impaired HIF-1α in the diabetic state, and up-regulate HIF-1α protein expression, further promote HIF-1/BNIP3-mediated mitochondrial autophagy and promptly remove dysfuction mitochondria, reduce ROS production, stabilize mitochondrial function, and thereby restore the protective effect of SPostC.

## Data Availability Statement

All datasets generated for this study are included in the article/supplementary material.

## Ethics Statement

This study was approved by the First Affiliated Hospital of Xinjiang Medical University Animal Ethics Committees.

## Author Contributions

Conceived and designed the experiments: HZ, LY, and TY. Performed the experiments: LY, JY, JJW, HM, and PX. Analyzed the data: JW, LY, XL, HW, JRY, and PX. Wrote the paper: LY, PX, HZ, HW, and JJW.

## Funding

This work was supported by the Key Laboratory Open Project of Xinjiang (Grant No. 2018D04001) of China.

## Conflict of Interest

The authors declare that the research was conducted in the absence of any commercial or financial relationships that could be construed as a potential conflict of interest.
